# Adenomatoid Tumor of the Testis: A Report of Two Cases and Literature Review

**DOI:** 10.7759/cureus.76809

**Published:** 2025-01-02

**Authors:** Amadadin Alhlib, Trudea Juma, Shahin Sayed

**Affiliations:** 1 Urology, The Aga Khan University, Nairobi, KEN; 2 Radiology, The Aga Khan University, Nairobi, KEN; 3 Pathology, The Aga Khan University, Nairobi, KEN

**Keywords:** adenomatoid tumor, benign tumor, testicular cancer, testicular mass, testicular tumor

## Abstract

Testicular adenomatoid tumor is a rare benign condition that can resemble testicular malignancies in its clinical presentation, potentially leading to overtreatment, such as radical orchidectomy. In our two case reports, we aim to better understand the behavior of this disease by examining its clinical presentation, as well as radiological, intraoperative, and pathological findings.

## Introduction

Adenomatoid tumors are benign mesenchymal tumors and can originate from the male genital system. They mainly represent 30% of para-testicular tumors that arise from the epididymis or spermatic cord [1,2]. Rarely do they present as intra-testicular tumors [2].

These conditions primarily affect middle-aged patients but can occur in any age group. Although the mesothelial origin of the tumor has been proven due to positive immunostaining of cytokeratin and vimentin, the risk factors and pathophysiology of the disease remain unclear [2]. Microscopically, testicular adenomatoid tumors display various morphologies, including acinar and solid variants [2].

The presenting features can mimic some testicular benign or malignant tumors [3]. Due to the lack of specific sonographic features, diagnosing the disease is primarily pathological. However, it can be clinically and sonographically suspected by the slowly growing tumor and the absence of surrounding tissue invasion. We are describing the clinical and sonographic features to avoid overtreating these lesions by doing radical orchidectomy.

## Case presentation

Case 1

Presentation

A 41-year-old male presented at the urology clinic with a complaint of a left testicular mass discovered two years ago. He reported pain on touch and gradual growth of the mass. The patient had no history of scrotal trauma, surgery, or infection, and no previous urogenital or extra-urogenital tuberculosis. Upon scrotal examination, a 1 cm left testicular nodule was found on the lateral side near the lower pole and adjacent to the epididymal tail. It was mildly tender and showed no skin changes. Both serum alpha-fetoprotein (AFP) and beta-human chorionic gonadotropin (β-HCG) levels were within the normal range.

Ultrasound of the Scrotum

**Figure 1:**
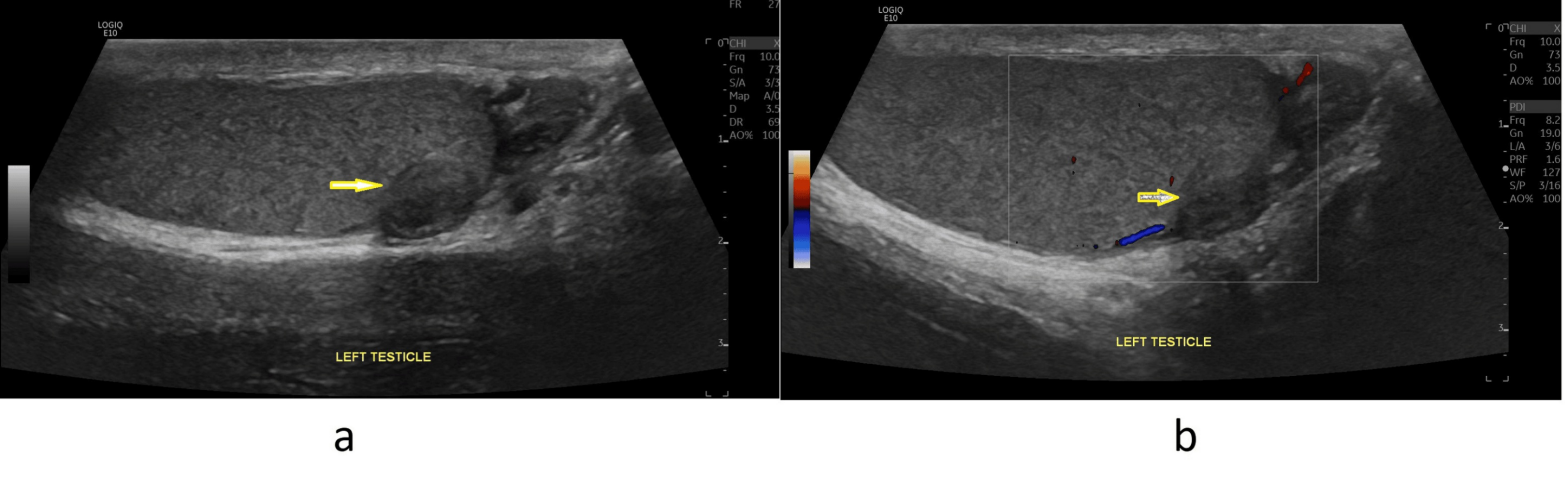
Left testicular mass. (a) Left testicular mass (yellow arrow). (b) Left testicular mass without increased vascularity (yellow arrow).

Left scrotal ultrasound images revealed a 1.0 x 0.8 cm mixed echogenicity mass at the lower pole of the left testicle with partially obscured margins and areas of acoustic shadowing suggesting intra-lesional calcifications (Figure 1A). No locally aggressive features were noted. There was no evidence of increased internal vascularity (Figure 1B).

Intraoperative Finding

The patient was taken to the operating room and placed under general anesthesia. Through the left inguinal approach, the left testicle was exposed and examined. A round firm nodule measuring 1 cm in diameter was found within the tunica albuginea. The mass did not invade surrounding structures or extend into the intratesticular tissue. A wide local excision of the mass was performed, and the edges of the tunica albuginea were sutured together.

Pathological Microscopic Finding

**Figure 2:**
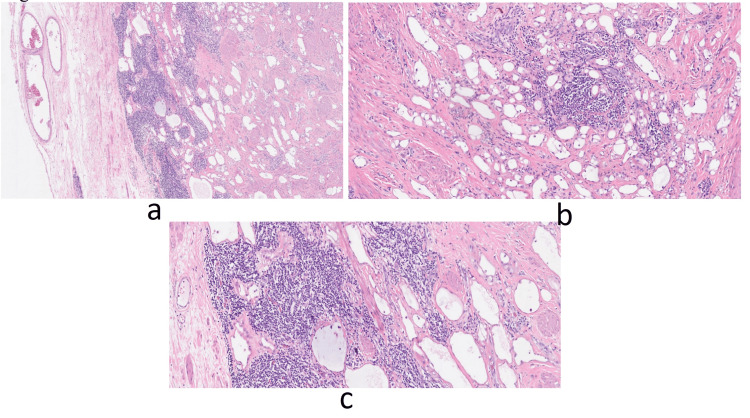
Pathology slides. (a-c) Pathological features of the left testicular adenomatoid tumor.

A specimen of the left testicular mass was submitted. The specimen consisted of a tan-white tissue fragment measuring 1 x 0.8 cm, which was all processed. Microscopically, the lesion was unencapsulated and composed of small tubulocystic spaces lined by cuboidal cells, with moderate to abundant eosinophilic cytoplasm containing single to multiple vacuoles. Signet ring cell morphology and lipoblast-like cells were also noted. There was no atypia or mitosis. Focal lymphoid aggregates were noted in the stroma. Benign seminiferous tubules with spermatogenesis were noted at the edge (Figure 2).

Case 2

Presentation

A 49-year-old male presented at the urology clinic with symptoms of urethritis and a right testicular mass was discovered during a genital examination. He mentioned that the mass was found 10 years ago, has been growing slowly, and is painless. He had no history of testicular trauma, infection, or surgery. The mass was 3 cm in diameter, irregular, non-tender, and inseparable from the lower right pole of the testis. Both serum AFP and β-HCG levels were within the normal range.

Ultrasound

**Figure 3:**
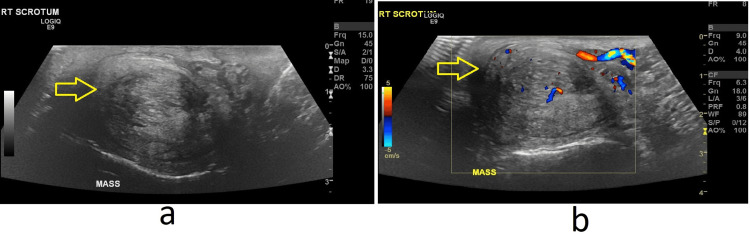
Right testicular mass (a) Right testicular mass (yellow arrow) (b) Right testicular mass with internal vascularity (yellow arrow)

Right scrotal ultrasound images showed a 3.2 x 2.2 cm circumscribed mixed echogenicity mass with a whorled appearance at the superior pole of the right testicle (Figure 3A). The right epididymis was not seen separate from the mass. A linear hypoechoic area separated the mass from the testicle, but no clear plane was noted. Trace fluid was present, and the spermatic cord/inguinal regions were clear (not shown in the images). Internal vascularity was present (Figure 3B).

Intraoperative Finding

The right inguinal approach was used while the patient was under general anesthesia to remove the testicle. The mass was nearly the same size as the testicle and was attached to the lower part of the right testicle. There was no clear plan for separation, but it did not invade the scrotal wall. A right radical orchidectomy was performed.

Pathological and Microscopic Findings

One well-circumscribed tumor with a well-defined border between the non-neoplastic testis and tumor was received. The tumor was solid, tan-white, and homogeneous with a whorled cut surface, without hemorrhage, necrosis, or cystic degeneration. The tumor measured 4 x 3 x 2.5 cm. It was seen to be confined to the tunica and did not extend beyond the tunica vaginalis. The spermatic cord, testicular hilum, and epididymis were not involved.

**Figure 4:**
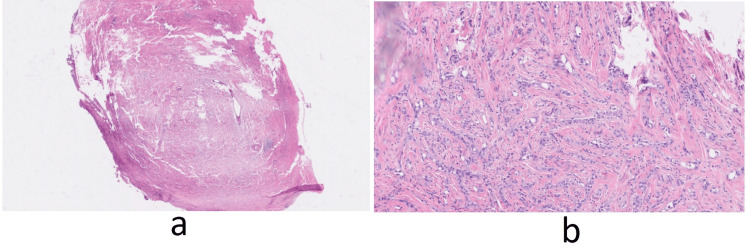
Pathology slides. (a and b) Pathological features of the right testicular adenomatoid tumor.

On microscopy, the tumor consisted of tubules, cords, and small nests lined by cuboidal cells with moderate to abundant eosinophilic to clear cytoplasm within a fibromuscular background. The testicular parenchyma showed focal atrophy. The lesion was completely excised (Figure 4).

## Discussion

Adenomatoid tumor is an uncommon mesothelial tumor that can affect both the male and female reproductive systems [1]. In men, it represents 30% of all paratesticular tumors and primarily originates from the parenchyma, potentially developing from the rete testis [2].

Most often, they are incidentally discovered as small (<2 cm), painless scrotal masses, but occasionally they can present as a painful mass [3]. They tend to grow gradually and can impact people of all ages, although they most frequently affect men in their 4th and 5th decades of life [3].

Adenomatoid tumors are benign and, due to the lack of specific clinical and radiological features, they are typically treated surgically using an inguinal approach [4]. This involves exploration with either radical or partial orchiectomy [4]. Surgical excision is generally curative with a low risk of recurrence [4].

Adenomatoid tumors of the scrotum are common extra-testicular tumors arising from the epididymis and less often the tunica (14%) [5]. Ultrasound is the initial imaging utilized to evaluate suspected scrotal masses making it imperative for the sonographer or radiologist to differentiate the epicenter of scrotal masses as extra- or intra-testicular. Evaluation of para-testicular tissues, vas deferens, and inguinal canals is key when a mass is found at initial imaging. In difficult cases, such as those arising from the visceral tunica, MRI correlation has the utility of determining the origin of the mass.

On imaging, adenomatoid tumors are small, circumscribed, ovoid, extra-testicular masses rarely larger than 5 cm. Sonographically, they have varied echogenicity often of similar echogenicity to the epididymis. However, cystic adenomatoid tumors have been described in the literature [6].

Our case reports demonstrate two cases of adenomatoid tumors, one presenting as an intra-testicular mass and the second with imaging characteristics mimicking an epidermoid cyst, both rare.

They present grossly as rounded nodules within the epididymis and consist of a proliferation of tubules and vascular-like spaces with varying morphologic appearance that range from cystically dilated spaces lined by attenuated mesothelium to glandular lumina lined by epithelioid cells [7]. Lymphoid aggregates may be seen as a company of the tumor [7]. Mitotic activity is rare. Diagnosis can be aided by immunoreactivity for pan-cytokeratin, CAM 5.2, CK7, calretinin, WT1, HBME-1, BAP1, and podoplanin [8].

Molecular studies demonstrate a somatic missense mutation in the TRAF7 gene, which encodes an E3 ubiquitin ligase and leads to the phosphorylation of nuclear factor-κB (NF-κB) [9]. Immunohistochemical expression of L1CAM highlights the TRAF7 mutation [9] and is a useful adjunct to diagnosis, especially in the differential consideration for both benign and malignant tumors such as lymphangioma [10] and metastatic signet ring carcinoma [11], in cases where intracytoplasmic vacuoles may be apparent.

## Conclusions

Testicular adenomatoid tumors are uncommon benign tumors that arise from spermatic cord structures, epididymis, or tunica albuginea. Diagnosis is only by histology because of the variety of clinical presentations and radiological features that make it impossible to diagnose it before excising. The similarity in most cases is slowly growing nodules and early excision would avoid radical orchiectomy.
